# Identification and Explanation of a Change in the Groundwater Regime using Time Series Analysis

**DOI:** 10.1111/gwat.12891

**Published:** 2019-05-10

**Authors:** Christophe Obergfell, Mark Bakker, Kees Maas

**Affiliations:** ^1^ Faculty of Civil Engineering and Geosciences Delft University of Technology Stevinweg 1, 2628 CN Delft The Netherlands; ^2^ Maas Geohydrologisch Advies Zuidsingel 114, 4331RW Middelburg The Netherlands

## Abstract

Time series analysis is applied to identify and analyze a transition in the groundwater regime in the aquifer below the sand ridge of Salland in the Netherlands, where groundwater regime refers to the range of head variations throughout the seasons. Standard time series analysis revealed a discrepancy between modeled and observed heads in several piezometers indicating a possible change in the groundwater regime. A new time series modeling approach is developed to simulate the transition from the initial regime to the altered regime. The transition is modeled as a weighted sum of two responses, one representing the initial state of the system, the other representing the altered state. The inferred timing and magnitude of the change provided strong evidence that the transition was the result of significant dredging works that increased the river bed conductance of the main river draining the aquifer. The plausibility of this explanation is corroborated by an analytical model. This case study and the developed approach to identify a change in the groundwater regime are meant to stimulate a more systematic application of time series analysis to detect and understand changes in groundwater systems which may easily go unnoticed in groundwater flow modeling.

## Introduction

The worldwide increase in groundwater demand requires increased care in assessing groundwater reserves, especially in the context of a changing climate (e.g., Wada et al. 2017). The awareness of the vulnerability of groundwater systems has motivated recent studies to better understand groundwater table dynamics and detect signs of overexploitation by searching for correlations between hydrological variables and climate forcings or land‐use changes (Stoll et al. 2011; Witte et al. 2015; Luo et al. 2016).

In groundwater hydrology, time series analysis has been applied to quantify decreasing trends in groundwater head (Weider and Boutt 2010), the effect of groundwater pumping (e.g., Baggelaar 1988; Van Geer et al. 1988; Von Asmuth et al. 2008; Harp and Vesselinov 2011; Obergfell et al. 2013; Shapoori et al. 2015) or to quantify the effects of river stage fluctuations (Barlow et al. 2000; Obergfell et al. 2016). The use of time series analysis to identify the effect of land‐use changes (e.g., Gehrels et al. 1994) or civil engineering interventions remains marginal, in spite of the sophistication of groundwater monitoring networks and the development of new analysis softwares (e.g., von Asmuth et al. 2012; Peterson and Western 2014; Collenteur et al. 2019).

The objective of this paper is to present a case study to demonstrate how time series analysis can be applied to identify and analyze a transition in the groundwater regime and help detect its cause. In this paper, the term groundwater regime refers to the range of head variations of a time series throughout the seasons.

The field site is a phreatic aquifer under a Pleistocene sand ridge in the Netherlands, where measured heads were indicative of a change in the regime. This paper is organized as follows. After a presentation of the field site, a standard time series analysis is presented, which clearly demonstrates a discrepancy between modeled and observed heads as the result of a regime change. A new modeling approach is then presented in which the response to recharge changes over time. The insights of the magnitude and timing of the change in the regime were the start of a search for a physical explanation. At the end of the paper, an explanation is found and an analysis is presented to corroborate the proposed explanation.

## Study Area

**Figure 1:**
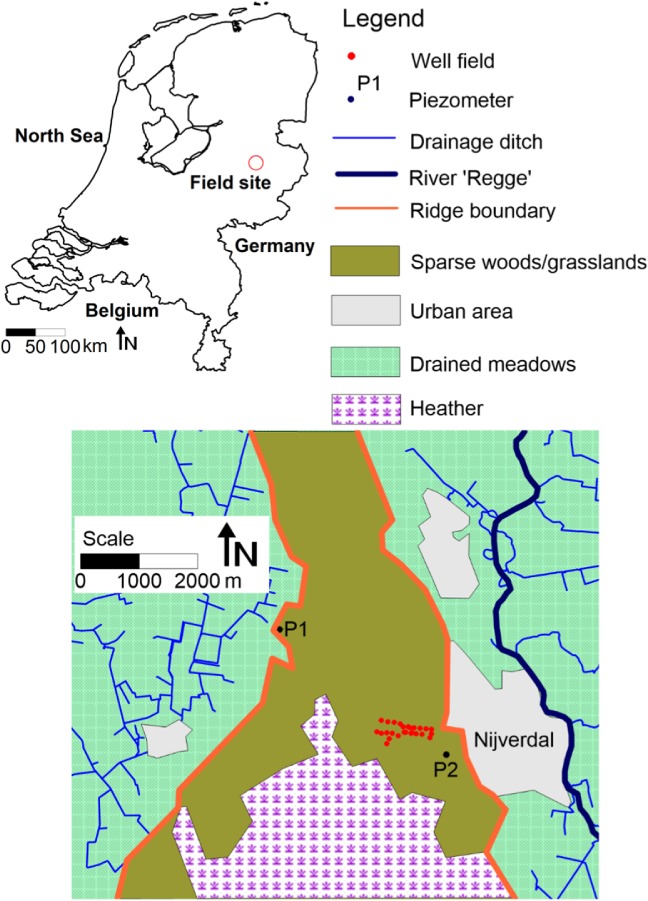
Field site at the ice‐pushed ridge of Salland.

The study area is located in the Eastern part of the Netherlands (Figure 1). The middle of the study area is formed by the ice‐pushed ridge of Salland which is approximately 12‐km long and 2‐ to 5‐km wide, with a maximum elevation of about 60 m Nieuwe Amsterdam Peil, (Dutch datum, approximately equal to mean sea level). The ridge built up at the margin of a glacier during the southernmost advance of the ice sheet in Northern Europe in the Pleistocene time (Saalian ice‐age, from about 200,000 to 130,000 years ago). The ridge consists of sand and the vegetation on the ridge consists of heather, grassland, deciduous trees, and coniferous woods.

Measurements at two representative piezometers in the area are used in this paper. Their characteristics are listed in Table 1. Piezometer 1 represents head fluctuations at a relatively short distance from draining ditches, with a fast reaction to recharge. Piezometer 2 represents head fluctuations on the sand ridge, with a much slower reaction to recharge (Figure 1). Heads were recorded twice a month.

**Table 1:** Description of Piezometers

Piezometer	Geological Survey ID	Screen Level (m NAP)	Surface Elevation (m NAP)	Median Observed Head (m NAP)
1	28AP0093	2.10	11.60	8.86
2	28CP0197	4.70	15.26	8.59

Note: ID = identification; NAP = Nieuwe Amsterdam Peil.

**Figure 2:**
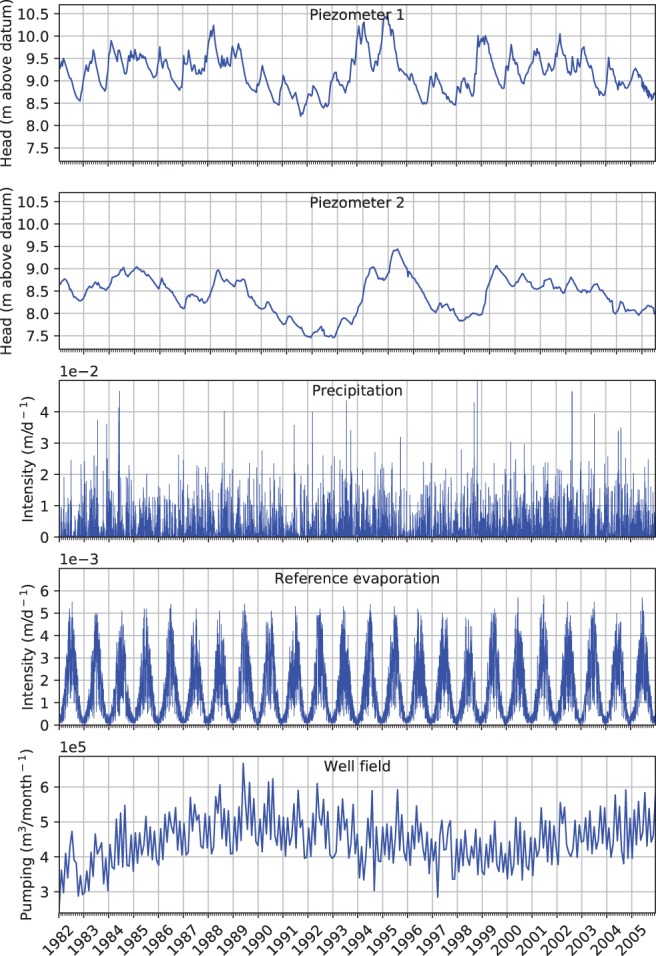
Time series of heads and stresses.

Daily precipitation and reference evaporation are obtained from the Royal Netherlands Meteorological Institute (The Royal Netherlands Meteorological Institute 2018). The reference evaporation is Makkink reference evaporation, defined as the evaporation of well‐watered short grass on a regional scale (Hooghart et al. 1988). Precipitation was measured at the meteorological station of Hellendoorn (Figure 1). Daily reference evaporation, which varies much less in space than precipitation, was measured at the meteorological station of de Bilt, about 85 km to the south‐west of the field site. A drinking water production well field is in operation on the ridge of Salland since 1954 (Figure 1). The average groundwater extraction is 5^.^10^6^ m^3^/year. The time series of the stresses are shown in Figure 2 and the location of the well field is shown in Figure 1.

## Time Series Modeling

### Standard Approach

As a first step, a standard time series model is fitted to the observed heads, using the method of predefined response functions (Von Asmuth et al. 2002). In this method, the head fluctuation *ϕ*(*t*) at an observation well, resulting from a stress applied on a groundwater system (precipitation, evaporation, or pumping) is obtained by convolution of the time series *q*(*t*)of the stress with a corresponding impulse response function *θ*(*t*):

(1) $$\phi \left(t\right)=\int_{0}^{t}q\left(\tau \right)\theta \left(t-\tau \right)d\tau$$

where *t* is time. The dependence of the response function on spatial coordinates is omitted in this notation. The response function *θ*(*t*) describes the reaction of the groundwater head to an instantaneous stress event of unit magnitude. In this paper, the scaled Gamma distribution is used as the impulse response function

(2) $$\theta \left(t\right)=M\frac{a^{n}t^{n-1}}{\Gamma \left(n\right)}e^{-\mathrm{at}}$$

where *M* is a scaling factor, *a* and *n* define the shape of the function, and Γ(*n*) is the Gamma function of *n*. The Gamma distribution is used frequently to simulate the response to recharge in time series models (e.g., Von Asmuth et al. 2002; Manzione et al. 2012; Obergfell et al. 2013; Peterson and Western 2014). The use of a Gamma distribution for the impulse response to recharge, including passage through the unsaturated zone was pioneered by Besbes and de Marsily (1984), and theoretically represents a succession of identical linear reservoirs (Nash 1957). The Gamma distribution is also applied here to simulate the slow response to pumping in a phreatic aquifer when piezometers are not in the direct vicinity of the pumping wells. Closer to the wells or in semiconfined aquifers, well functions such as Hantush may be more appropriate as pumping response functions (e.g., Von Asmuth et al. 2008; Obergfell et al. 2013).

The mean *μ* and standard deviation *σ*of the response function are related to parameters *a* and *n* as (Weisstein 2018a):

(3) $$\mu =\frac{n}{a}$$

(4) $$\sigma =\frac{\sqrt{n}}{a}$$

The observed heads are modeled as the sum of the response to recharge, the response to pumping and a reference level *d*:

(5) $$h_{o}=\phi_{r}+\phi_{w}+d+r$$

where *r*(*t*)is the remaining residual, and *ϕ*_*r*_ is the response to recharge *R*:

(6) $$\phi_{r}\left(t\right)=\int_{0}^{t}R\left(\tau \right)\theta_{r}\left(t-\tau \right)d\tau$$

where *θ*_*r*_is the impulse response function of recharge. The recharge in Equation 6 is approximated as (e.g., Von Asmuth et al. 2002)

(7) $$R\left(\tau \right)=P\left(\tau \right)-\mathrm{fE}\left(\tau \right)$$

where *P*(*τ*) is the measured precipitation, *E*(*τ*) is the measured reference evaporation, and *f* is a scaling factor. Runoff is neglected given the absence of streams on the ridge, the high permeability of the soil, the flat to gently sloping relief of the sand ridge and its surroundings, and the moderate climate (Meinardi et al. 1998). The term *ϕ*_*w*_ in Equation 5 is the response to pumping discharge *Q*(*τ*):

(8) $$\phi_{w}=\int_{0}^{t}Q\left(\tau \right)\theta_{w}\left(t-\tau \right)d\tau$$

where *θ*_*w*_ is the impulse response function of pumping. Modeling the residuals with an exponential decay process transforms the time series of residuals into a noise time series *n*(*t*) that is approximately white (Von Asmuth and Bierkens 2005). The residual at time *t*_*i*_is related to the residual at time *t*_*i* − 1_as

(9) $$r\left(t_{i}\right)=r\left(t_{i-1}\right)\exp\left(-\alpha \left(t_{i}-t_{i-1}\right)\right)+n\left(t_{i}\right)$$

where *α* is the residual decay factor and *n*(*t*_*i*_)is the remaining noise at time *t*_*i*_.

The observed heads and stresses are written with respect to their arithmetic means over the considered time period, so that Equation 5 becomes

(10) $$\begin{array}{cc} h_{o}-{\bar{h}}_{o} & =\left(\phi_{r}-{\bar{\phi }}_{r}\right)+\left(\phi_{w}-{\bar{\phi }}_{w}\right)\\ & +\left({\bar{\phi }}_{r}+{\bar{\phi }}_{w}\right)-{\bar{h}}_{o}+d+r \end{array}$$

where the overbar indicates the arithmetic mean. The drainage base is set equal to the mean observed head ${\bar{h}}_{o}$ minus the sum of the mean contributions of the stresses (similar to Von Asmuth et al. 2002)

(11) $$d={\bar{h}}_{o}-\left({\bar{\phi }}_{r}+{\bar{\phi }}_{w}\right)$$

so that

(12) $$h_{o}-{\bar{h}}_{o}=\left(\phi_{r}-{\bar{\phi }}_{r}\right)+\left(\phi_{w}-{\bar{\phi }}_{w}\right)+r$$

The mean response ${\bar{\phi }}_{r}$is computed, using (2) as the response function, as

(13) $${\bar{\phi }}_{r}=\bar{R}\int_{0}^{\infty }\theta_{r}\left(t-\tau \right)d\tau =\bar{R}M_{r}$$

*M*_*r*_ is the final response of the groundwater head when the recharge is applied continuously with unit intensity. Similarly, ${\bar{\phi }}_{w}=\bar{Q}M_{w}$.

Parameter optimization is performed by minimizing the objective function *S* (**p**) defined as half of the sum of the squared noise terms *n*_*i*_defined in Equation 9:

(14) $$S\left(p\right)=\frac{1}{2}\sum_{i=1}^{N_{o}}n_{i}^{2}$$

where **p** is the vector of *N*_*p*_ log‐transformed model parameters and *N*_*o*_ is the number of observations. The search for the minimum of the objective function was performed using a modified Gauss‐Newton algorithm (e.g., Hill 1998). The covariance matrix **C** of the optimized parameters is approximated as (e.g., Carrera and Neuman 1986; Yuen 2010):

(15) $$C≃\sigma^{2}H^{-1}≃\sigma^{2}{\left(J^{T}J\right)}^{-1}$$

where **H** is the Hessian of the objective function, **J** is the Jacobian matrix, and *σ* ^2^is the sample variance of the noise

(16) $$\sigma^{2}=\frac{1}{N_{o}}\sum_{i=1}^{N_{o}}{\left(n_{i}-\bar{n}\right)}^{2}$$

where $\bar{n}$ is the arithmetic mean of the noise.

In the parameter optimization process, all parameters are log‐scaled except for the drainage base to prevent parameters going negative during the estimation process. The optimum is used as the starting point for a second optimization without log‐scaled parameters to compute a covariance matrix.

## Results of Standard Time Series Analysis

**Figure 3:**
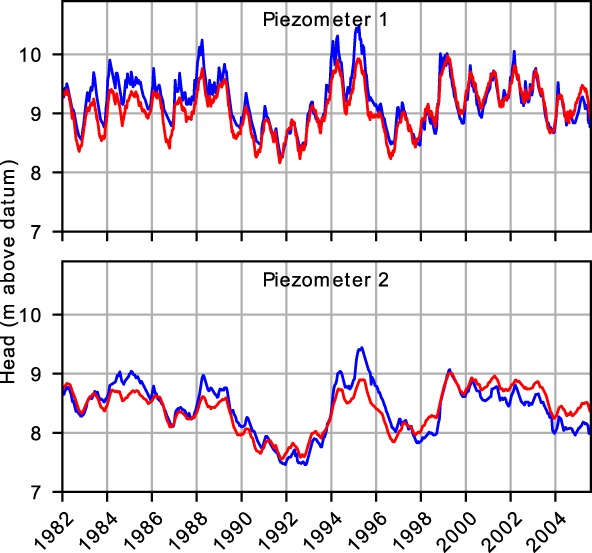
Observed heads (blue) and modeled heads (red) over the period 1982 to 2005, standard time series analysis.

Standard time series analysis is applied to the entire observation period from 1982 to 2005. The model parameters for the standard model consist of five parameters *M*_*r*_, *a*_*r*_, and *n*_*r*_ of the response function to recharge, the factor *f* and the exponential noise decay factor *α*. For Piezometer 2, three additional parameters are optimized: the parameters *M*_*w*_, *a*_*w*_, and *n*_*w*_of the response function to pumping. For Piezometer 1, the effect of pumping could not be identified. The goodness of fit of the time series models is expressed as the Nash‐Sutcliffe coefficient (Nash and Sutcliffe 1970) defined as

(17) $$\mathrm{NS}=1-\frac{\sum_{i=1}^{N_{o}}{\left(h_{m,i}-h_{o,i}\right)}^{2}}{\sum_{i=1}^{N_{o}}{\left(h_{o,i}-{\bar{h}}_{o}\right)}^{2}}$$

where *h*_*m*, *i*_is the modeled head at time *i* and *h*_*o*, *i*_ is the observed head at time *i*. The observed and modeled heads for Piezometers 1 and 2 are shown in Figure 3. The blue line corresponds to the observed head and the red line corresponds to the best fit obtained over the whole period 1982 to 2005. The Nash‐Sutcliffe coefficient is 77.3% for Piezometer 1 and 77.2% for Piezometer 2.

The models that are fitted over the entire period 1982 to 2005 structurally underestimate the observed heads before 1997 and overestimate the observed heads after 1997. One of the reasons for the poor fit could be a linear decreasing trend resulting from the intensification of groundwater use and land‐use changes in the Netherlands (Witte et al. 2015). Fitting a trend to the observed time series only results in a slight improvement of the fit for Piezometer 1 but not for both piezometers, so this hypothesis is rejected.

**Figure 4:**
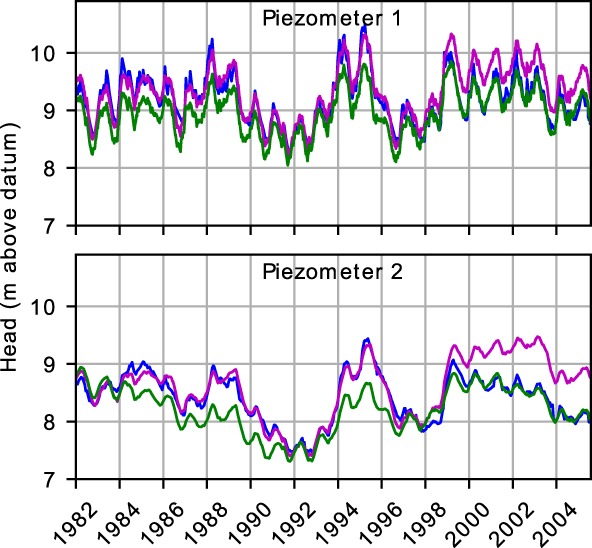
Observed heads in Piezometers 1 and 2 (blue), models calibrated over the period 1982 to 1997 (magenta) and models calibrated over the period 1997 to 2005 (green).

As a next step in the investigation, the model is fitted separately over the period 1982 to 1997 and over the period 1997 to 2005 (Figure 4). In both cases, excellent fits are obtained over the calibration period but the model structurally departs from the observed heads outside the calibration period. The model calibrated over the period 1982 to 1997 clearly overestimates the observed heads after 1997 and the model calibrated over the period 1997 to 2005 clearly underestimates the observed heads before 1997.

Further analysis showed that no trend in the precipitation or evaporation time series can be detected that may contribute to the observed head decline. The only significant reported land‐use change that affects the recharge rate is an ecohydrological project involving the yearly conversion of 100 ha of woods into heather. This project aimed at restoring wetter conditions as heather reportedly results in lower evaporation than woods. However, this project started after 2001 and cannot explain a change in the regime in the mid 1990s. Furthermore, this intervention is expected to result in a rising trend of the heads while the opposite is observed. Regarding other factors, no increase of pumping or decline of stream stages were reported in the mid 1990s. At this point, it is concluded that the groundwater regime over the period 1982 to 1997 underwent a substantial change in the middle of the 1990s.

### Modeling of the Transition Period

A new method is proposed to simulate the transition from one system response to another system response (possible physical reasons for such a transition are discussed in the next sections). The main idea is to replace the response to recharge by a weighted sum of two responses, one representing the initial state and the other representing the altered state.

The transition from one response to another response is implemented as follows. Suppose that the response function changes from *θ*_1_(*t*) to *θ*_2_(*t*). Convolution of these response functions with the recharge time series results in two different responses, *ϕ*_1_(*t*) and *ϕ*_2_(*t*). The transition from the first to the second state can be described by a weighted sum of the two responses:

(18) $$\phi \left(t\right)=\omega \left(t\right)\phi_{1}\left(t\right)+\left(1-\omega \left(t\right)\right)\phi_{2}\left(t\right)$$

where *ϕ*(*t*) is the weighted sum of the two responses and *ω*(*t*) is the weighting function. The weighing function *ω*(*t*) is chosen to be an S‐shaped curve that varies from 1 to 0:

(19) $$\omega \left(t_{i}\right)=\frac{1}{e^{\beta \left(t-\sigma \right)}+1}$$

where *β* is a shape factor that determines the smoothness of the transition and *σ*is the middle of the transition time, when the function takes a value of 0.5.

**Figure 5:**
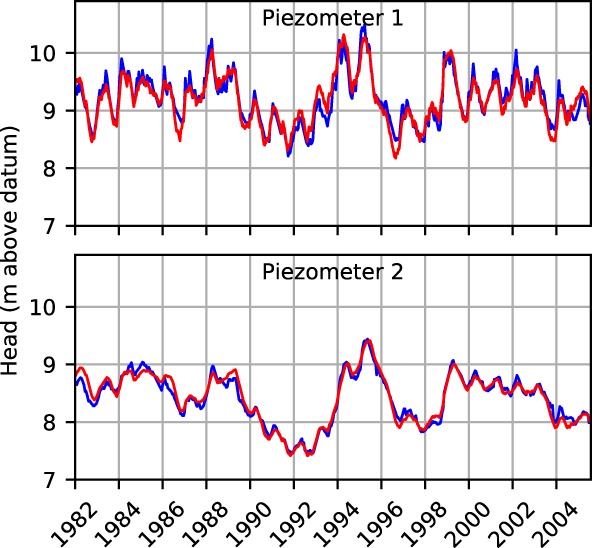
Fit obtained with a weighted sum of responses to recharge. Observed head (blue) and modeled head (red).

A new time series model is fitted to the entire observation period. Nine parameters are fitted for Piezometer 1: *M*_*r*_, *a*_*r*_, and *n*_*r*_ of the response function to recharge before the transition, *M*_*r*_, *a*_*r*_, and *n*_*r*_ of the response function to recharge after the transition, the factor *f*, the exponential noise decay factor *α*, and the parameter *σ* of the transition function (19). The solution is not sensitive to the sharpness of the transition for Piezometer 1 so that the shape factor *β* was fixed to 50 which corresponds to a sharp transition. For Piezometer 2, parameters *M*_*w*_, *a*_*w*_, and *n*_*w*_ of the response function to pumping are also included for a total of 13 parameters. The new model gives a good fit for the entire measurement period for both piezometers (Figure 5). The Nash‐Sutcliffe has increased from 75.3% to 93.5% for Piezometer 1 and from 66.4% to 95.5% for Piezometer 2. The sum of squared residuals has decreased by 73% for Piezometer 1 and by 87% for Piezometer 2. An improvement of the fit was expected, of course, as the complexity of the model, and hence the number of parameters, was increased. The question arises whether the improvement of the fit is significant enough to justify the increase in complexity. One way to answer this question is to compare the values of the Akaike information criterium (AIC) for the model residuals (e.g., Banks and Joyner 2017, Equation 6):

(20) $$\mathrm{AIC}=\left(2k+1\right)+N\log\left(S/N\right)$$

where *k* is the number of parameters, *N* is number of data points, and *S* is the sum of squared residuals. The AIC drops from −1889 to −2690 for Piezometer 1 and from −1629 to −2775 for Piezometer 2, indicating that the additional complexity of including the transition in regime is justified. It is noted, however, that the residuals are correlated, while the AIC theory is developed assuming uncorrelated residuals. A second evaluation of the AIC was performed with a time interval between residuals which is sufficiently long to consider the resulting residuals uncorrelated (based on the noise decay factor). For Piezometer 1, a time interval of 4 months leads to approximately uncorrelated residuals while for Piezometer 2, a time interval of 23 months must be used. With approximately uncorrelated residuals, the AIC drops from −229 to −306 for Piezometer 1 and from −25 to −55 for Piezometer 2, which confirms that using the model with the regime transition is justified.

**Figure 6:**
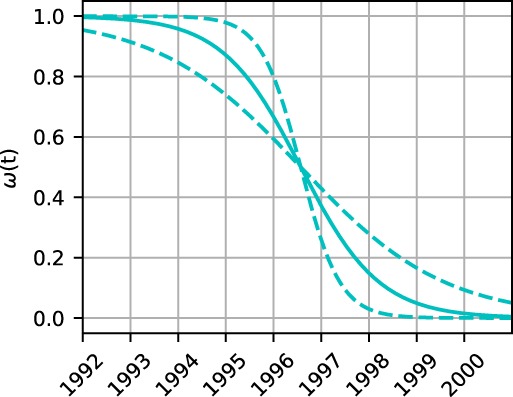
Weighting function for Piezometers 2 with 95% confidence limits (dotted lines); the transition time corresponds to the time at which the weighing function takes the value 0.5.

The transition time was identified with reasonable confidence intervals for both piezometers. The sharpness of the transition was determined with statistical significance for Piezometer 2 and was fixed for Piezometer 1, as stated. The resulting weighting function for Piezometer 2, including the 95% limits, is shown in Figure 6.

**Figure 7:**
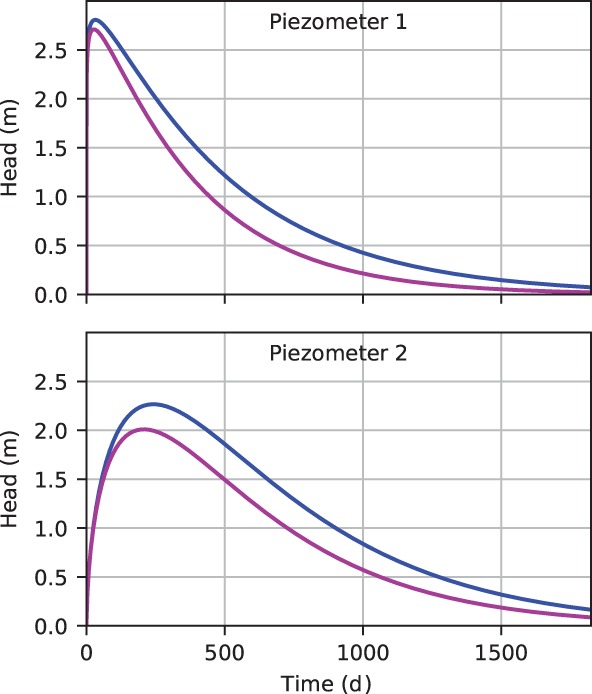
Comparison of the impulse response functions before (blue) and after the transition (red) for Piezometer 1 and Piezometer 2; Time zero corresponds to the time of a short recharge event of unit magnitude.

The response functions that prevails before and after the transition are shown in Figure 7. The corresponding parameters are shown in Table 2 in terms of *M*_*r*_, *μ*_*r*_, and *σ*_*r*_ .

**Table 2:** Comparison of the Parameters of the Response Function to Recharge That Prevails Before and After the System Transition Time; Numbers in Brackets Are Lower and Upper Limits of the 95% Confidence Intervals

	Piezometer 1 Before Transition	Piezometer 1 After Transition	Piezometer 2 Before Transition	Piezometer 2 After Transition	
*M*_*r*_ × 10e^3^	1.59 [1.54,1.64]	1.18 [1.15,1.21]	2.06 [2.03,2.09]	1.60 [1.58,1.62]
*μ* × 10e^2^	4.89 [4.7,5.08]	3.69 [3.53,3.87]	6.50 [6.43,6.56]	5.16 [5.10,5.21]
*σ* × 10e^2^	4.71 [4.50,4.92]	3.57 [3.37,3.78]	5.15 [5.08,5.21]	4.06 [3.99,4.11]

The quicker recession of the response to recharge after the transition indicates that groundwater is drained faster. The next step is to search for an underlying physical reason.

### Physical Explanation

The local waterboard reported that significant dredging works had been carried out in the river the “Regge” (Figure 1) between 1992 and 1994 as part of an environmental cleanup project. Archived documents showed that 250,000 m^3^ of sediments (contaminated with heavy metals) were removed from the river bed over a distance of 8 km. A probably unintentional consequence was an increase of the river bed conductance and thus of the draining capacity of the river. This explanation is consistent with the reduction of the response to recharge shown in Figure 7.

**Figure 8:**
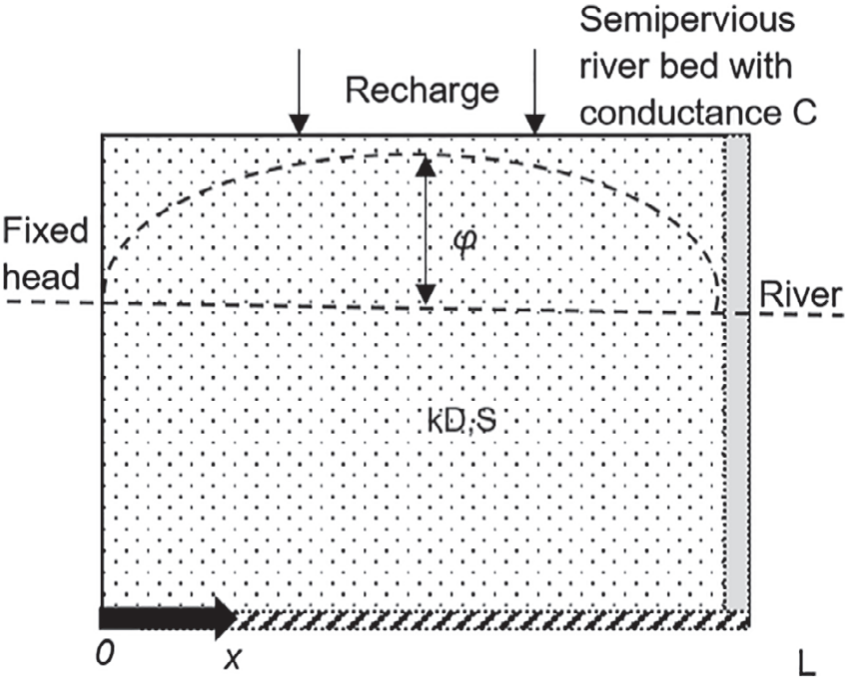
Simplified cross‐section of study area.

As a final step in the investigation, a short theoretical analysis is presented to determine whether a change in the river bed conductance can indeed result in the change in the response function presented in Table 2. A simplified East‐West cross‐section over the sand ridge is shown in Figure 8. The hydraulic conductivity is *k*, approximate saturated thickness is *D*, storage coefficient is *S*and length of the cross‐section is *L*. The aquifer is bounded on the left by a fixed head representing the drained meadows to the west of the study area. The aquifer is bounded to the right by a river representing the river “Regge.”

Flow into the stream is computed as:

(21) $$Q_{s}=C\phi \left(x=L\right)$$

where *Q*_*s*_ [L^2^/T] is the flux from the aquifer to the river per unit length of river, *ϕ*(*x* = *L*) is the head in the aquifer relative to the stream stage at the semipervious stream bank, and *C*is the river bed conductance [L/T]. The base of the aquifer is impermeable and aquifer parameters are uniform and constant. Heads are measured with respect to the heads on the left and right sides of the cross‐section (which are equal). Initially, the head is zero everywhere. The analytical solution for the response function due to a constant recharge starting at time *t* = 0 is given by Bruggeman (1999), Equation 137‐48). The response function *θ*(*t*) is characterized by its moments of order *k* defined as:

(22) $$M_{k}=\int_{0}^{\infty }t^{k}\theta \left(t\right)\mathrm{dt}$$

The three first moments are considered here and evaluated by numerical integration of the solution of Bruggeman. Alternatively, the moments can be obtained by solving the differential equation for the moments of the response function (e.g., Bakker et al. 2007; Carr and Simpson 2018).

The moments are recombined and expressed as the mean response time *μ* and the standard deviation*σ*of the response function (Weisstein 2018b):

(23) $$\mu =\frac{M_{1}}{M_{0}}$$

(24) $$\sigma^{2}=\frac{M_{2}}{M_{0}}-\mu^{2}$$

In the simplified model, precipitation reaches the groundwater table instantaneously, which implies that the time it takes for infiltrated water to percolate through the unsaturated zone is neglected. This approximation does not affect the estimation of parameter *M*_*r*_ which determines the final response to recharge. Values obtained for this parameter with the analytical solution should therefore correspond approximately to the values obtained with the time series model. In contrast, neglecting the passage through the unsaturated zone results in a smaller mean response time *μ*_*r*_ and standard deviation *σ*_*r*_. However, the change of these two parameters as a result of variations of the river bed conductance remains comparable to the time series model.

**Figure 9:**
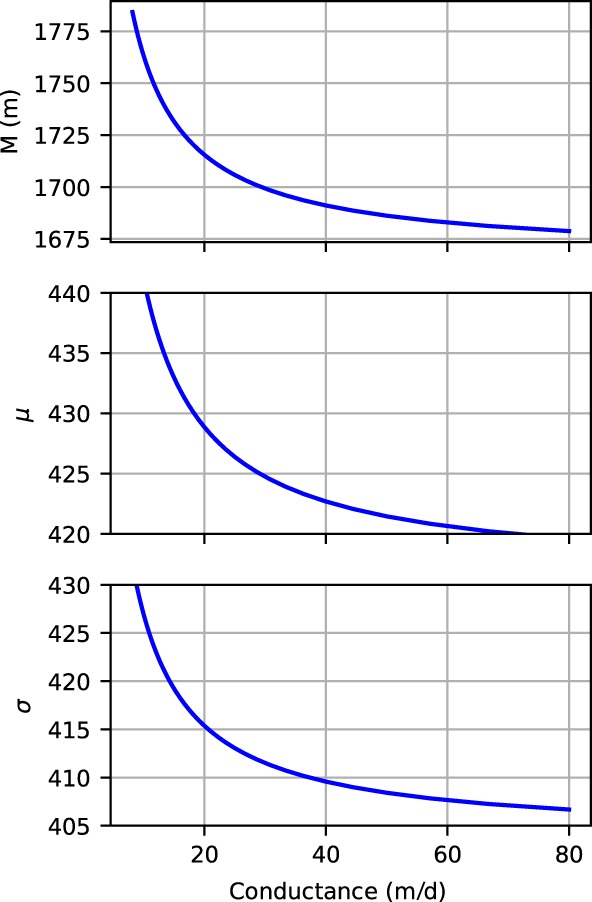
Influence of the river bed conductance on the characteristics of the impulse response function to recharge.

Values that approximate the situation of Piezometer 2 are chosen with k = 40 m/d, *D* = 30 m, *S* = 0.3, and L = 4000 m. The values corresponding to the magnitude of the final response to precipitation *M*_*r*_, the mean time *μ*_*r*_ and standard deviation *σ*_*r*_ are evaluated for river bed conductance values *C* ranging from 10 to 80 m/d, at a distance *x* = *L*/2. The results are plotted in Figure 9.

An increase of the river bed conductance from *C*= 10 m/d to *C*= 50 m/d leads to a theoretical reduction of approximately 5% to 10% of the parameters *M*_*r*_, *μ*_*r*_, and *σ*_*r*_ of the response function to recharge. These reductions support the conjecture that the observed transition in groundwater regime is the result of dredging works that increased the conductance of the river bed.

## Conclusion

In this study, it is shown how time series analysis can be used as an investigative tool to identify and analyze changes in the groundwater regime that are otherwise unnoticed. Standard time series analysis revealed a systematic discrepancy between modeled and observed heads in the phreatic aquifer under the sand ridge of Salland in the Netherlands. The investigated piezometers exhibit two different fluctuation regimes that can be modeled accurately when considered separately. A new time series modeling approach is developed that incorporates a weighted sum of two modeled responses to recharge, one representing the initial state and the other representing the altered state. The new approach results in excellent fits of the heads over the entire observation period. The results of the new approach initiated a search for the physical causes of the regime change, leading to the conclusion that dredging works in the river draining the aquifer are most likely the cause of the groundwater regime change. The local water board was not aware of this change to the groundwater regime and the change had not been incorporated in any of the groundwater models used for water management purposes.

Finally, it is pointed out that changes in climate, land use, or groundwater pumping, which are the usual suspects when changes in head variations are observed, do not appear to be involved here. Time series analysis of observed heads is an instrumental tool to understand the history of the dynamics of an aquifer and deserves a more systematic application in the analysis of groundwater systems.
